# Impact of Resting Heart Rate on Cardiovascular Mortality According to Serum Albumin Levels in a 24-year Follow-up Study on a General Japanese Population: NIPPON DATA80

**DOI:** 10.2188/jea.JE20210114

**Published:** 2023-05-05

**Authors:** Yiwei Liu, Aya Hirata, Tomonori Okamura, Daisuke Sugiyama, Takumi Hirata, Aya Kadota, Keiko Kondo, Takayoshi Ohkubo, Katsuyuki Miura, Akira Okayama, Hirotsugu Ueshima

**Affiliations:** 1 Department of Preventive Medicine and Public Health, Keio University School of Medicine, Tokyo, Japan; 2 Faculty of Nursing and Medical Care, Keio University, Kanagawa, Japan; 3 Department of Public Health, Hokkaido University Faculty of Medicine, Sapporo, Japan; 4 Department of Public Health, Shiga University of Medical Science, Shiga, Japan; 5 Department of Hygiene and Public Health, Teikyo University School of Medicine, Tokyo, Japan; 6 Center for Epidemiologic Research in Asia, Shiga University of Medical Science, Shiga, Japan; 7 Research Institute of Strategy for Prevention, Tokyo, Japan

**Keywords:** cardiovascular disease, mortality, serum albumin, resting heart rate, cohort study

## Abstract

**Background:**

Elevated resting heart rate (RHR) is associated with an increased risk of cardiovascular disease (CVD) and all-cause mortality. However, the findings of cohort studies differed. Thus, the impact of RHR on CVD mortality might be different according to the background of the population. Therefore, we examined the relationship of RHR and CVD mortality according to serum albumin (ALB) levels in a Japanese general population.

**Methods:**

In total, 8,363 individuals without a history of CVD were followed for 24.0 years. The participants were divided into four groups according to the quartiles of RHR (Q1–Q4), and they were further classified into the high and low ALB groups based on a median value of 44 g/L. We estimated the multivariable-adjusted hazard ratios (HRs) of CVD mortality in each RHR group based on ALB levels, and the interaction between RHR and ALB groups on CVD mortality was evaluated.

**Results:**

We found no significant association between RHR and CVD mortality. However, the Q4 of RHR was significantly associated with an increased risk for CVD mortality (HR 1.27; 95% confidence interval [CI], 1.02–1.57) in participants with a low ALB level. Meanwhile, the Q4 of RHR was significantly correlated with a decreased risk for CVD morality in those with a high ALB level (HR 0.61; 95% CI, 0.47–0.79) after adjusting for covariates. A significant interaction between RHR and ALB for CVD mortality was shown (*P* < 0.001).

**Conclusion:**

The impact of RHR on CVD mortality differed according to ALB levels in a general Japanese population.

## INTRODUCTION

Elevated resting heart rate (RHR) is associated with an increased risk of coronary heart disease (CHD)^1^^,^^2^ heart failure (HF),^3^^,^^4^ cardiovascular disease (CVD),^5^^,^^6^ and all-cause mortality^7^^,^^8^ in the general population. This association is observed in all age groups in different clinical settings, irrespective of other atherosclerotic risk factors.^9^^,^^10^ However, the mechanism underlying the relationship between RHR and cause-specific mortality or morbidity remains unknown. To date, clinical trials have not shown the importance of slow RHR in hypertensive patients without CVD.^10^^,^^11^ Accordingly, comorbidities underlying a high RHR should be further validated. A high RHR is an index of increased sympathetic nervous system activity,^12^ which might be associated with the development of arrythmia, arterial stiffness, and hypertension. In the general population, sympathetic nervous system activity is commonly accelerated by individual responses to continuous mental stress.^13^ Further, lack of regular aerobic exercise, smoking, and heavy alcohol drinking are associated with a high RHR.^14^

Serum albumin (ALB) is a nutritional factor influencing general health status.^15^ Moreover, it has anti-inflammatory, antioxidant, anticoagulant, and antiplatelet aggregation properties. From a mechanism perspective, ALB is able to combine with nitric oxide (NO)^16^ to maintain a normal level. ALB is also involved in recovery from ischemia injury.^17^ ALB, even within the clinical normal range, can significantly influence the incidence of CVD and all-cause mortality.^18^ Such subclinical inflammation might increase RHR and decrease heart-rate variability, ultimately leading to mortality.^19^ A previous study has shown that nutritional intake influences RHR variability.^20^ However, to the best of our knowledge, the association of ALB level and RHR with mortality has not been comprehensively assessed. To validate this notion, we conducted a long-term cohort study to assess the impact of RHR on cause-specific mortality according to ALB levels.

## METHODS

### Study participants

We performed a cohort study based on the National Survey on Circulatory Disorders in 1980, which was referred to as the National Integrated Project for Prospective Observation of Non-communicable Diseases and Its Trends in the Aged, 1980 (NIPPON DATA 80). Details of the cohort are provided elsewhere.^21^^–^^23^ In brief, we randomly selected 300 districts in Japan, and a total of 10,546 participants (4,640 men and 5,906 women) aged >30 years participated in the baseline survey, with a participation rate of 77%. Details of baseline examinations and follow-up surveys are provided in eMaterials 1.

Of 10,546 participants, 2,183 were excluded due to the following reasons: history of CVD (*n* = 676), missing information at baseline survey (*n* = 598), and lost to follow-up because of incomplete access to residential information (*n* = 909). There was no selection bias in terms of baseline characteristics between participants with and without follow-up. Finally, 8,363 participants (3,666 men and 4,697 women) were included in the analysis.

### Ethical approval

The current study was conducted in accordance with the ethical guidelines of Shiga University of Medical Science (R2005-021) and Keio University School of Medicine (2018-0108).

### Statistical analysis

The participants were divided into four groups according to RHR (beats/min) quartiles (Q1–Q4) as follows: Q1, <62; Q2, 62–68; Q3, 69–77; and Q4, >77 beats/min in all participants; Q1, <60; Q2, 60–66; Q3, 67–73; and Q4, >73 beats in men; and Q1, <64; Q2, 64–70; Q3, 71–77; and Q4, >77 beats in women. The mean values and standard deviation were presented as continuous variables, and the number and proportion as categorical variables according to each RHR category. Continuous variables were compared between groups using one-way analysis of variance, and categorical variables were compared using the χ2-test. We assessed the age- and multivariable-adjusted hazard ratios (HRs) and 95% confidence intervals (CIs) of each RHR category for all-cause death, total CVD death, cause-specific CVD mortality, and non-CVD mortality using a Cox proportional hazards model. Model 1 was adjusted for age; model 2 for the variable in model 1 plus body mass index, blood glucose levels, systolic blood pressure, hypertension treatment, total cholesterol level, smoking status, and alcohol drinking status; and model 3 for the variables in model 2 plus ALB level. Then, sex was adjusted in the sex-combined analysis. Further, we estimated the HRs of RHR quartiles according to ALB levels based on the median values (low: <44 and high: ≥44 g/L) for outcomes using models 1 and 2. In addition, we generated the interaction term by multiplying RHR as a continuous variable and ALB groups, and evaluated the interaction on all-cause, CVD, and non-CVD mortality in model 2 using a Cox proportional hazards. To validate the influence of arrhythmia on HR, we performed the same analysis with exclusion of individuals with atrial fibrillation or frequent supraventricular and/or ventricular premature beats. Two-sided *P*-values of <0.05 were considered statistically significant. For baseline characteristics, we used one-way ANOVA to test if covariates were distributed equally between groups. Statistical analysis was performed with the R package version 3.6.1 (R Foundation for Statistical Computing, Vienna, Austria).

## RESULTS

The characteristics of participants according to RHR quantiles at the baseline survey are shown in Table 1. The mean non-fasting blood glucose levels were high in both men and women with a high RHR. The proportion of individuals receiving hypertension treatment was highest among women in the group with the lowest RHR.

**Table 1.** Characteristics of study participants according to resting heart rate quartiles at baseline survey in 1980

Total (Quantiles in beats/min)	Q1 (<62)	Q2 (62–68)	Q3 (69–77)	Q4 (>77)	*P* value
*N*	2,225	2,049	2,138	1,951	—
Age, years	51 (13)	49 (13)	49 (13)	50 (14)	<0.001
Sex, male, %	59	44	37	34	<0.001
Smoking, %	33	29	26	26	<0.001
Drinking, %	39	38	35	32	<0.001
Body mass index, kg/m^2^	22 (2.9)	23 (3.1)	23 (3.5)	23 (3.5)	0.01
Total cholesterol, mmol/L	4.7 (0.8)	4.7 (0.9)	4.7 (0.9)	4.8 (0.8)	<0.001
Non-fasting blood glucose, mmol/L	6.9 (1.6)	7.1 (1.9)	7.2 (1.8)	7.7 (2.4)	<0.001
Systolic blood pressure, mm Hg	138 (23)	135 (22)	136 (23)	139 (22)	<0.001
Serum albumin, g/L	44 (2.6)	44 (2.6)	44 (2.6)	44 (2.8)	0.04
Hypertension treatment, %	15.1	14	11	9.6	0.09

Men (Quantiles in beats/min)	Q1 (<60)	Q2 (60–66)	Q3 (67–73)	Q4 (>73)	*P* value

*N*	903	910	945	908	—
Age, years	51 (13)	50 (13)	49 (13)	50 (13)	0.08
Smoking, %	56.3	63.3	64.9	67.1	<0.001
Drinking, %	73.3	75.1	74.1	75.8	0.82
Body mass index, kg/m^2^	22 (2.8)	22 (2.7)	23 (2.8)	23 (3.1)	<0.001
Total cholesterol, mmol/L	4.8 (0.8)	4.8 (0.9)	4.9 (0.8)	5.0 (0.9)	<0.001
Non-fasting blood glucose, mmol/L	6.9 (1.7)	7.1 (2.0)	7.3 (1.8)	7.8 (2.6)	<0.001
Systolic blood pressure, mm Hg	135 (21)	137 (20)	139 (21)	143 (21)	<0.001
Serum albumin, g/L	44 (3.1)	44 (3.0)	45 (3.0)	44 (3.0)	0.002
Hypertension treatment, %	9.2	10.8	8.5	10.9	0.19

Women (Quantiles in beats/min)	Q1 (<64)	Q2 (64–70)	Q3 (71–77)	Q4 (>77)	*P* value

*N*	1,273	1,151	1,102	1,171	—
Age, years	52 (13)	50 (13)	50 (14)	50 (14)	<0.001
Smoking, %	7.3	8.1	10.2	9.2	0.21
Drinking, %	20.9	20.8	20.5	17.7	0.33
Body mass index, kg/m^2^	23 (3.0)	23 (3.4)	23 (3.5)	23 (3.6)	0.87
Total cholesterol, mmol/L	4.8 (0.9)	4.9 (0.8)	4.9 (0.9)	5.0 (1.0)	0.49
Non-fasting blood glucose, mmol/L	6.9 (1.5)	7.1 (1.6)	7.2 (1.9)	7.6 (2.3)	<0.001
Systolic blood pressure, mm Hg	132 (22)	131 (21)	134 (21)	137 (21)	<0.001
Serum albumin, g/L	43 (2.2)	44 (2.3)	44 (2.9)	44 (2.9)	<0.001
Hypertension treatment, %	14.7	10.2	10.3	9.9	<0.001

Values reported as mean (standard deviation) unless otherwise noted.

During a mean follow-up of 24.0 years, with 203,021 person-years of observation time, the total number of deaths was 3,128 (1,055 CVD deaths and 2,073 non-CVD deaths). Among CVD deaths, 219 were caused by CHD and 455 by stroke. The crude mortality rates and HRs for all-cause, CVD, and non-CVD mortality according to RHR quartiles are presented in Table 2. Compared with Q1, Q4 was significantly associated with an increased risk for all-cause mortality after adjusting for confounders in all participants (HR 1.15; 95% CI, 1.04–1.28). In addition, Q4 was significantly associated with an increased risk for non-CVD mortality in both men and women (HR 1.21; 95% CI, 1.03–1.41 vs HR 1.19; 95% CI, 1.00–1.43). Meanwhile, RHR was not significantly associated with an increased risk for CVD mortality. ALB level was significantly associated with CVD mortality (HR 0.49; 95% CI, 0.38–0.65).

**Table 2.** Crude mortality rates and hazard ratios for all-cause, cardiovascular disease (CVD) and non-CVD mortality according to resting heart rate (RHR) quartiles

All-cause mortality					

Total	Quantiles (beats/min)	Q1 (<62)	Q2 (62–68)	Q3 (69–77)	Q4 (>77)

	Number of participants	2,225	2,049	2,138	1,951
	Person-years	53,526	51,022	52,743	46,836
	Number of events	892	733	750	753
	Crude mortality	16.6	14.4	14.2	16.1
	Hazard ratio				
	Model 1	1	1.07 [0.97, 1.18]	1.07 [0.97, 1.19]	1.21 [1.10, 1.34]^***^
	Model 2	1	1.03 [0.94, 1.14]	1.03 [0.93, 1.14]	1.11 [1.00, 1.23]^*^
	Model 3	1	1.04 [0.94, 1.15]	1.04 [0.95, 1.15]	1.15 [1.04, 1.28]^*^

Men	Quantiles (beats/min)	Q1 (<60)	Q2 (60–66)	Q3 (67–73)	Q4 (>73)

	Number of participants	1,044	920	794	908
	Person-years	24,376	21,914	19,057	20,579
	Number of events	591	375	327	338
	Crude mortality	24.2	17.1	17.1	16.4
	Hazard ratio				
	Model 1	1	1.05 [0.92, 1.21]	0.99 [0.86, 1.14]	1.21 [1.06, 1.37]^**^
	Model 2	1	0.98 [0.86, 1.12]	0.95 [0.82, 1.09]	1.10 [0.96, 1.25]
	Model 3	1	1.01 [0.88, 1.15]	0.96 [0.83, 1.10]	1.13 [0.99, 1.29]

Women	Quantiles (beats/min)	Q1 (<64)	Q2 (64–70)	Q3 (71–77)	Q4 (>77)

	Number of participants	1,273	1,151	1,102	1,171
	Person-years	32,002	29,402	27,637	29,159
	Number of events	414	346	357	380
	Crude mortality	12.9	11.7	12.9	13.0
	Hazard ratio				
	Model 1	1	1.05 [0.90, 1.21]	1.08 [0.94, 1.24]	1.16 [1.01, 1.34]^*^
	Model 2	1	1.03 [0.89, 1.19]	1.05 [0.91, 1.21]	1.09 [0.94, 1.26]
	Model 3	1	1.05 [0.91, 1.21]	1.09 [0.95, 1.26]	1.15 [0.99, 1.33]

CVD mortality					

Total	Quantiles (beats/min)	Q1 (<62)	Q2 (62–68)	Q3 (69–77)	Q4 (>77)

	Number of participants	2,225	2,049	2,138	1,951
	Person-years	53,526	51,022	52,743	46,836
	Number of events	315	230	260	250
	Crude mortality	5.9	4.5	4.9	5.3
	Hazard ratio				
	Model 1	1	0.93 [0.78, 1.10]	1.02 [0.86, 1.20]	1.07 [0.91, 1.27]
	Model 2	1	0.87 [0.74, 1.04]	0.94 [0.80, 1.11]	0.93 [0.78, 1.10]
	Model 3	1	0.88 [0.74, 1.05]	0.96 [0.81, 1.14]	0.98 [0.82, 1.16]

Men	Quantiles (beats/min)	Q1 (<60)	Q2 (60–66)	Q3 (67–73)	Q4 (>73)

	Number of participants	1,044	920	794	908
	Person-years	24,376	21,914	19,057	20,579
	Number of events	149	124	99	132
	Crude mortality	6.1	5.7	5.2	6.4
	Hazard ratio				
	Model 1	1	1.07 [0.84. 1.36]	0.99 [0.77, 1.28]	1.14 [0.90, 1.45]
	Model 2	1	0.96 [0.76, 1.22]	0.90 [0.70, 1.17]	0.96 [0.75, 1.22]
	Model 3	1	0.98 [0.77, 1.25]	0.92 [0.71, 1.18]	0.99 [0.78, 1.26]

Women	Quantiles (beats/min)	Q1 (<64)	Q2 (64–70)	Q3 (71–77)	Q4 (>77)

	Number of participants	1,273	1,151	1,102	1,171
	Person-years	32,002	29,402	27,637	29,159
	Number of events	158	123	131	139
	Crude mortality	4.9	4.2	4.7	4.8
	Hazard ratio				
	Model 1	1	0.98 [0.77, 1.24]	1.01 [0.80, 1.27]	1.08 [0.86, 1.36]
	Model 2	1	0.97 [0.76, 1.23]	0.99 [0.78, 1.26]	0.99 [0.78, 1.26]
	Model 3	1	0.99 [0.78, 1.26]	1.05 [0.83, 1.33]	1.08 [0.85, 1.37]

Non-CVD mortality					

Total	Quantiles (beats/min)	Q1 (<62)	Q2 (62–68)	Q3 (69–77)	Q4 (>77)

	Number of participants	2,225	2,049	2,138	1,951
	Person-years	53,526	51,022	52,743	46,836
	Number of events	577	503	490	503
	Crude mortality	10.8	9.9	9.3	10.7
	Hazard ratio				
	Model 1	1	1.15 [1.02, 1.29]^*^	1.10 [0.98, 1.25]	1.28 [1.14, 1.45]^***^
	Model 2	1	1.12 [0.99, 1.27]	1.07 [0.95, 1.21]	1.22 [1.08, 1.38]^**^
	Model 3	1	1.13 [1.01, 1.28]^*^	1.09 [0.96, 1.23]	1.26 [1.11, 1.42]^***^

Men	Quantiles (beats/min)	Q1 (<60)	Q2 (60–66)	Q3 (67–73)	Q4 (>73)

	Number of participants	1,044	920	794	908
	Person-years	24,376	21,914	19,057	20,579
	Number of events	324	270	221	312
	Crude mortality	13.3	12.3	11.6	15.2
	Hazard ratio				
	Model 1	1	1.05 [0.89, 1.23]	0.99 [0.83, 1.17]	1.24 [1.06, 1.44]^**^
	Model 2	1	0.99 [0.85, 1.17]	0.96 [0.81, 1.14]	1.18 [1.01, 1.38]^*^
	Model 3	1	1.02 [0.86, 1.20]	0.98 [0.82, 1.16]	1.21 [1.03, 1.41]^*^

Women	Quantiles (beats/min)	Q1 (<64)	Q2 (64–70)	Q3 (71–77)	Q4 (>77)

	Number of participants	1,273	1,151	1,102	1,171
	Person-years	32,002	29,402	27,637	29,159
	Number of events	256	223	226	241
	Crude mortality	8.0	7.6	8.2	8.3
	Hazard ratio				
	Model 1	1	1.08 [0.91, 1.30]	1.12 [0.94, 1.34]	1.20 [1.01, 1.43]^*^
	Model 2	1	1.07 [0.89, 1.28]	1.09 [0.91, 1.30]	1.14 [0.95, 1.37]
	Model 3	1	1.08 [0.90, 1.30]	1.12 [0.93, 1.34]	1.19 [1.00, 1.43]^*^


Model 1 is adjusted for gender (in gender specific model no gender adjusted) and age.

Model 2 is adjusted for gender, age, body mass index (BMI), blood glucose, systolic blood pressure (SBP), hypertension treatment, total-cholesterol, smoking status, and alcohol drinking status.

Model 3 is adjusted for gender, age, BMI, serum albumin (ALB), blood glucose, SBP, hypertension treatment, total-cholesterol, smoking status, and alcohol drinking status.

^*^P < 0.05 ^**^P < 0.01 ^***^P < 0.001

The crude mortality rates and HRs for all-cause, CVD, and non-CVD mortality according to RHR quartiles in the low and high ALB level groups are depicted in Table 3. In the low ALB level group, compared with Q1, Q4 was significantly associated with an increased risk for all-cause mortality after adjusting for confounders in all participants and women (HR 1.35; 95% CI, 1.18–1.53 vs HR 1.24; 95% CI, 1.03–1.49). Moreover, a correlation was observed between Q4 and an increased risk for CVD mortality in the low ALB level group (HR 1.27; 95% CI, 1.02–1.57). Meanwhile, Q4 was significantly associated with a decreased risk for CVD morality in all participants in the high ALB level group (HR 0.61; 95% CI, 0.47–0.79). Moreover, Q4 was significantly correlated with an increased risk for non-CVD mortality in all participants in the low ALB level group (HR 1.27; 95% CI, 1.07–1.50). A significant interaction between RHR and ALB for CVD mortality was shown (*P* < 0.001); however, such an interaction was not evident for non-CVD mortality (*P* = 0.31). After excluding individuals with atrial fibrillation (*n* = 57) and frequent supraventricular and/or ventricular premature beats (*n* = 99), or those who received antihypertensive treatment, the results remained did not change significantly (data not shown).

**Table 3.** Crude mortality rates and hazard ratios for all-cause, cardiovascular disease (CVD) and non-CVD mortality according to resting heart rate (RHR) quantiles in low and high serum albumin (ALB)

**All-cause mortality**	Low albumin				High albumin				

Total (RHR Quantile in beats/min)	Q1 (<62)	Q2 (62–68)	Q3 (69–77)	Q4 (>77)	Q1 (<62)	Q2 (62–68)	Q3 (69–77)	Q4 (>77)	*P*-values for interaction
	
Number of participants	1,007	896	910	797	1,218	1,153	1,228	1,154	
Person-years	21,936	31,589	20,661	30,361	20,863	31,879	17,253	29,582	
Number of deaths	533	418	427	411	359	315	323	342	
Crude mortality rate/1,000 person-years	24.3	13.2	20.7	13.5	17.2	9.9	18.7	11.6	
Hazard ratio									
Model 1	1	0.82 [0.72, 0.94]^**^	0.83 [0.73, 0.94]^**^	0.98 [0.86, 1.11]	0.45 [0.39, 0.51]^***^	0.40 [0.35, 0.47]^***^	0.40 [0.34, 0.45]^***^	0.45 [0.40, 0.52]^***^	
Model 2	1	1.02 [0.89, 1.16]	1.12 [0.99, 1.28]	1.35 [1.18, 1.53]^***^	0.85 [0.74, 0.97]^*^	0.98 [0.85, 1.13]	0.88 [0.77, 1.02]	0.96 [0.83, 1.10]	*P* = 0.007

Men (RHR Quantile in beats/min)	Q1 (<60)	Q2 (60–66)	Q3 (67–73)	Q4 (>73)	Q1 (<60)	Q2 (60–66)	Q3 (67–73)	Q4 (>73)	
	
Number of participants	424	328	270	325	620	592	524	583	
Person-years (total)	8,350	6,483	5,486	6,048	16,025	15,430	13,570	14,530	
Number of deaths	283	220	175	234	190	174	145	210	
Crude mortality rate/1,000 person-years	33.9	33.9	31.9	38.7	11.9	11.3	10.7	14.5	
Hazard ratio									
Model 1	1	1.05 [0.88, 1.25]	0.99 [0.82, 1.20]	1.26 [1.06, 1.50]^**^	0.78 [0.64, 0.94]^**^	0.84 [0.69, 1.02]	0.78 [0.64, 0.96]^*^	0.94 [0.78, 1.12]	
Model 2	1	0.99 [0.83, 1.18]	0.94 [0.78, 1.14]	1.18 [0.99, 1.40]	0.85 [0.69, 1.02]	0.83 [0.68, 1.02]	0.81 [0.65, 0.99]^*^	0.90 [0.74, 1.08]	*P* = 0.25

Women (RHR Quantile in beats/min)	Q1 (<64)	Q2 (64–70)	Q3 (71–77)	Q4 (>77)	Q1 (<64)	Q2 (64–70)	Q3 (71–77)	Q4 (>77)	
	
Number of participants	670	581	504	508	603	570	598	663	
Person-years (total)	16,065	14,380	12,081	11,818	15,936	15,022	15,555	17,340	
Number of deaths	257	204	202	214	157	142	155	166	
Crude mortality rate/1,000 person-years	16.0	14.2	16.7	18.1	9.9	9.5	10.0	9.6	
Hazard ratio									
Model 1	1	1.03 [0.85, 1.24]	1.12 [0.93, 1.35]	1.29 [1.08, 1.55]^**^	0.88 [0.72, 1.07]	0.95 [0.77, 1.17]	0.93 [0.76, 1.14]	0.93 [0.77, 1.13]	
Model 2	1	1.03 [0.86, 1.23]	1.11 [0.92, 1.34]	1.24 [1.03, 1.49]^*^	0.89 [0.73, 1.09]	0.95 [0.77, 1.16]	0.90 [0.74, 1.11]	0.87 [0.71, 1.06]	*P* = 0.01

**CVD mortality**	Low albumin				High albumin				

Total (RHR Quantile in beats/min)	Q1 (<62)	Q2 (62–68)	Q3 (69–77)	Q4 (>77)	Q1 (<62)	Q2 (62–68)	Q3 (69–77)	Q4 (>77)	
	
Number of participants	1,007	896	910	797	1,218	1,153	1,228	1,154	
Person-years (total)	21,936	31,589	20,661	30,361	20,863	31,879	17,253	29,582	
Number of deaths	193	139	141	159	122	91	119	91	
Crude mortality rate/1,000 person-years	8.8	4.4	6.8	5.2	5.8	2.9	6.9	3.1	
Hazard ratio									
Model 1	1	0.91 [0.73, 1.13]	0.99 [0.80, 1.23]	1.35 [1.09, 1.67]^**^	0.92 [0.73, 1.16]	0.89 [0.69, 1.16]	0.98 [0.77, 1.23]	0.75 [0.58, 0.96]^*^	
Model 2	1	0.87 [0.70, 1.09]	0.95 [0.76, 1.19]	1.27 [1.02, 1.57]^*^	0.95 [0.76, 1.20]	0.83 [0.64, 1.07]	0.88 [0.70, 1.12]	0.61 [0.47, 0.79]^***^	*P* < 0.001

Men (RHR Quantile in beats/min)	Q1 (<60)	Q2 (60–66)	Q3 (67–73)	Q4 (>73)	Q1 (<60)	Q2 (60–66)	Q3 (67–73)	Q4 (>73)	
	
Number of participants	424	328	270	325	620	592	524	583	
Person-years (total)	8,350	6,483	5,486	6,048	16,025	15,430	13,570	14,530	
Number of deaths	283	220	175	234	190	174	145	210	
Crude mortality rate/1,000 person-years	33.9	33.9	31.9	38.7	11.9	11.3	10.7	14.5	
Hazard ratio									
Model 1	1	1.14 [0.84, 1.55]	0.92 [0.65, 1.30]	1.32 [0.97, 1.79]	0.93 [0.67, 1.30]	0.92 [0.64, 1.31]	1.01 [0.71, 1.45]	0.91 [0.65, 1.28]	
Model 2	1	1.07 [0.78, 1.45]	0.85 [0.60, 1.20]	1.18 [0.87, 1.61]	1.00 [0.71, 1.41]	0.84 [0.59, 1.21]	0.97 [0.67, 1.40]	0.75 [0.53, 1.07]	*P* = 0.25

Women (RHR Quantile in beats/min)	Q1 (<64)	Q2 (64–70)	Q3 (71–77)	Q4 (>77)	Q1 (<64)	Q2 (64–70)	Q3 (71–77)	Q4 (>77)	
	
Number of participants	670	581	504	508	603	570	598	663	
Person-years (total)	16,065	14,380	12,081	11,818	15,936	15,022	15,555	17,340	
Number of deaths	257	204	202	214	157	142	155	166	
Crude mortality rate/1,000 person-years	16.0	14.2	16.7	18.1	9.9	9.5	10.0	9.6	
Hazard ratio									
Model 1	1	0.97 [0.72, 1.31]	0.96 [0.71, 1.30]	1.32 [0.99, 1.75]	0.85 [0.62, 1.19]	0.86 [0.61, 1.22]	0.95 [0.69, 1.31]	0.72 [0.51, 1.02]	
Model 2	1	0.97 [0.72, 1.32]	0.98 [0.72, 1.33]	1.32 [0.98, 1.76]	0.83 [0.60, 1.16]	0.82 [0.58, 1.17]	0.88 [0.64, 1.22]	0.59 [0.42, 0.85]^**^	*P* = 0.002

**Non-CVD death**	Low albumin				High albumin				

Total (RHR Quantile in beats/min)	Q1 (<62)	Q2 (62–68)	Q3 (69–77)	Q4 (>77)	Q1 (<62)	Q2 (62–68)	Q3 (69–77)	Q4 (>77)	
	
Number of participants	1,007	896	910	797	1,218	1,153	1,228	1,154	
Person-years (total)	21,936	31,589	20,661	30,361	20,863	31,879	17,253	29,582	
Number of deaths	340	279	286	252	237	224	204	251	
Crude mortality rate/1,000 person-years	15.5	8.8	13.8	8.3	11.4	7.0	11.8	8.5	
Hazard ratio									
Model 1	1	1.08 [0.92, 1.27]	1.20 [1.02, 1.40]^*^	1.33 [1.13, 1.56]^***^	0.82 [0.69, 0.97]^*^	1.02 [0.86, 1.21]	0.83 [0.70, 0.99]^*^	1.06 [0.90, 1.25]	
Model 2	1	1.07 [0.91, 1.25]	1.16 [0.99, 1.36]	1.27 [1.07, 1.50]^**^	0.87 [0.73, 1.03]	1.05 [0.88, 1.25]	0.85 [0.71, 1.02]	1.05 [0.89, 1.24]	*P* = 0.31

Men (RHR Quantile in beats/min)	Q1 (<60)	Q2 (60–66)	Q3 (67–73)	Q4 (>73)	Q1 (<60)	Q2 (60–66)	Q3 (67–73)	Q4 (>73)	
	
Number of participants	424	328	270	325	620	592	524	583	
Person-years (total)	8,350	6,483	5,486	6,048	16,025	15,430	13,570	14,530	
Number of deaths	89	75	51	77	60	49	48	55	
Crude mortality rate/1,000 person-years	10.7	11.6	9.3	12.7	3.7	3.2	3.5	3.8	
Hazard ratio									
Model 1	1	1.01 [0.81, 1.25]	1.02 [0.82, 1.28]	1.24 [1.01, 1.53]^*^	0.72 [0.57, 0.90]^**^	0.80 [0.63, 1.01]	0.70 [0.54, 0.90]^**^	0.94 [0.75, 1.16]	
Model 2	1	0.96 [0.77, 1.20]	0.99 [0.78, 1.24]	1.18 [0.95, 1.46]	0.79 [0.62, 0.99]^*^	0.83 [0.66, 1.05]	0.75 [0.58, 0.97]^*^	0.97 [0.77, 1.21]	*P* = 0.52

Women (RHR Quantile in beats/min)	Q1 (<64)	Q2 (64–70)	Q3 (71–77)	Q4 (>77)	Q1 (<64)	Q2 (64–70)	Q3 (71–77)	Q4 (>77)	
	
Number of participants	670	581	504	508	603	570	598	663	
Person-years (total)	16,065	14,380	12,081	11,818	15,936	15,022	15,555	17,340	
Number of deaths	155	128	131	123	101	95	95	118	
Crude mortality rate/1,000 person-years	9.6	8.9	10.8	10.4	6.3	6.3	6.1	6.8	
Hazard ratio									
Model 1	1	1.07 [0.84, 1.35]	1.22 [0.96, 1.54]	1.25 [0.99, 1.59]	0.89 [0.70, 1.15]	1.01 [0.78, 1.30]	0.92 [0.71, 1.19]	1.06 [0.83, 1.35]	
Model 2	1	1.05 [0.83, 1.33]	1.19 [0.94, 1.51]	1.18 [0.93, 1.50]	0.93 [0.73, 1.20]	1.02 [0.79, 1.33]	0.92 [0.71, 1.19]	1.05 [0.82, 1.34]	*P* = 0.46

Model 1 is adjusted for gender (in gender specific model no gender adjusted) and age.

Model 2 is adjusted for gender, age, body mass index (BMI), blood glucose, systolic blood pressure (SBP), hypertension treatment, total-cholesterol, smoking status, and alcohol drinking status.

*P*-values for the interaction were those between resting heart rate (continuous) and serum albumin groups.

^*^P < 0.05 ^**^P < 0.01 ^***^P < 0.001

## DISCUSSION

The current study found no significant association between RHR and CVD mortality. In the analysis stratified according to ALB levels, a higher RHR was significantly associated with an increased risk for CVD mortality among individuals with low ALB levels. Meanwhile, in those with high ALB levels, a higher RHR was correlated with a lower risk for CVD mortality. A positive association was observed between RHR and non-CVD mortality, and a higher RHR was significantly associated with a greater risk among individuals with low ALB levels. However, this result was not observed among individuals with high ALB levels. Thus, the effect of RHR on outcomes, particular on CVD mortality, differed according to ALB levels.

In the current study, we did not observe a direct association between a high RHR and CVD mortality in all participants. In previous cohort studies, meanwhile, RHR was found to be associated with an increased risk for CVD and all-cause mortality in the general population.^10^^,^^11^^,^^24^ As we previously reported, an elevated RHR was found to be correlated with cardiovascular or all-cause mortality in a 16.5-year follow-up. However, it was only observed in participants aged 30–59 years.^23^ In previous cohort studies on a Japanese general population with a mean age of about 45 years at baseline, a higher RHR was associated with the development of obesity and diabetes within 20 years^25^ and hypertension within 3 years.^26^ These findings suggest that the activation of the sympathetic nervous system correlated with a high RHR might play a role in the development of these conditions in relatively younger populations. Furthermore, participants with an activated sympathetic nervous system may develop adverse events in the early follow-up period.

Regarding the effect of reducing RHR, previous studies have reported that all-cause mortality can be reduced in patients with HF by lowering RHR with the use of medications, such as beta-blockers.^27^ However, no findings showed the additional value of RHR-lowering therapy with beta-blockers in hypertensive patients without CVD.^10^^,^^11^^,^^24^ In a consensus conference wherein recommendations for the management of hypertensive patients with increased RHR were updated, gathering panelists reconfirmed that RHR-lowering therapy had no additional benefit for tachycardia hypertension. They recommended that further research should be performed to validate unresolved discrepancies between the findings of cohort studies and clinical trials.^28^ Accordingly, the effect of RHR on mortality is controversial, which might be caused by the characteristics of participants.

In our analysis stratified according to ALB levels, a higher RHR was significantly associated with CVD mortality and non-CVD mortality among individuals with low ALB levels. Since ALB is a marker of anti-inflammatory, antioxidant, anticoagulant, and antiplatelet aggregation activities, as well as nutritional status, it might be correlated with the risk for mortality.^29^ In the present study, individuals with lower ALB levels could present with subclinical changes in health status, although they might not experience actual malnutrition. However, the protein intake proportion showed no significant difference between ALB groups (mean 15.30; standard deviation [SD], 2.12 g/1,000 kcal in low ALB vs mean 15.27; SD, 2.10 g/1,000 kcal in high ALB) in the present study, a significant difference was shown in total energy intake (mean 2,063; SD, 491 kcal/day in low ALB vs mean 2,193; SD, 510 kcal/day in high ALB, *P* < 0.001). Hypoalbuminemia is associated with an increased risk of death in HF patients with reduced ejection fraction, in whom the magnitude of heart rate reduction is associated with the survival benefit of beta-blockers.^30^ Moreover, ALB is associated with myocardial fibrosis, adverse pulsatile aortic hemodynamics, and prognosis in HF patients with preserved ejection fraction.^31^ There are individuals with latent HF even in the general population.^32^ ALB has protective effects against low-density lipoprotein cholesterol from Cu2+ oxidation, which is a crucial step for atherogenesis, in addition to HF.^33^ Glycated or oxidated ALB can no longer hold copper ion.^34^ Abnormal ALB levels can decrease the ALB/high-sensitivity C-reactive protein ratio, and this proinflammation imbalance can result in the development or worsening of coronary slow flow.^35^ Overall, a normal ALB level is crucial for preventing CVD, and low ALB levels caused by high oxidative levels and glycation stress disrupt anti-atherogenesis functioning. Such a strong effect might account for the impact of RHR. Accordingly, individuals with health impairment might be at risk for a high RHR, which is considered a marker of mortality. Also, if we take a deeper subgroup analysis for cause-specific mortality in CVD, the cerebral infarction made a great contribution to it (eTable 2). In this analysis, ALB level differently impacted males and females according to RHR quantile, which could explain why RHR quantile elevation caused both increase and decrease in ALB in the stratified CVD mortality model. This is similar to a previous study in Japan.^36^

On the contrary, in the present study, a lower risk in higher RHR was shown among individuals with high ALB. This result might be controversial compared to previous evidence. Actually, there is a little physiological evidence supporting our results. Some studies have shown that lower RHR was associated with left ventricular hypertrophy (LVH) progression.^37^^,^^38^ LVH is a risk factor for CVD events and all-cause mortality even in a general population. The present study might suggest that lower RHR among individuals with high ALB increases risk of CVD mortality mediated by potential LVH progression. We conducted an additional analysis considering recorded findings of left high R-wave (l-high-R) on electrocardiogram, which was performed as the National Survey on Circulatory Disorders in 1980. Participants with lower RHR had higher proportion of those with l-high-R. However, the association between RHR and CVD mortality was almost unchanged after adjusting for l-high-R, irrespective of ALB levels. Another explanation is that, while elevated heart rate was associated with increased peripheral blood pressure, there was an inverse relationship between resting heart rate and augmentation index,^39^ and elevated augmentation index and consequently increased central blood pressure were risk factors for CVD events.^40^^,^^41^ Particularly, the inverse association was more apparent in higher level of aortic stiffness among normotensive and untreated hypertensive participants.^42^ Furthermore, in the CAFÉ study,^37^ the use of a beta-blocker was associated with RHR reduction and central aortic pressure elevation simultaneously, despite similar brachial systolic blood pressure. These findings suggest that RHR might reflect central hemodynamics, which relate to CVD events. Additionally, when we take a closer look to cause-specific CVD mortality influenced by RHR at high ALB, they were mostly from CHD and cerebral infarction, sequelae of atherosclerotic cardiovascular disease. As described earlier, ALB has anti-atherosclerotic properties, such as anti-inflammatory and antioxidant effects, as well as being a marker of nutritional status; therefore, individuals with relatively higher ALB would be protected against atherosclerosis. Accordingly, we speculate that RHR at high ALB has a different impact on CVD from that of low ALB. However, there is little evidence on the relationship between RHR and ALB, which may include many unexamined confounding factors, such as socioeconomic status and mental stress. Although the background on the results is not clear, we believe our results are important because this is a first finding, at least to our knowledge. Future studies are needed to clarify the clinical significance of RHR.

The current study had several limitations. First, we can only adjust some basic confounders in mortality models. Other potential risk factors, such as thyroid hormone function, physical activity, high-density lipoprotein cholesterol, and inflammatory marker levels could not be adjusted in the statistical models. Second, since this study was based on risk factor measurement on one occasion only, our findings did not reflect changes in RHR and confounding risk factors, such as blood pressure and serum glucose level during follow-up. Third, we did not have a precise information on the treatment of hypertension; thus, participants who used beta-blockers were not identified. Finally, we measured RHR via electrocardiogram during daytime, and data on RHR obtained during nighttime or daytime immediately after waking were not available. A follow-up study on six populations in several countries, including Japan, used the RHR obtained during office measurements, and data on 24-h ambulatory blood pressure were used. Results showed that morning and nighttime, not daytime, RHRs were associated with CVD mortality.^37^

### Conclusion

The impact of RHR on CVD mortality differed according to ALB levels in a general Japanese population. Such an effect might depend on health status, including nutrition. Thus, further studies must be conducted to elucidate the underlying mechanism.

## SUPPLEMENTARY MATERIAL

eTable 1. Baseline characteristics for participants

eTable 2. Crude mortality rates and hazard ratios for CHD, stroke, cerebral infarction and cerebral hemorrhage mortality according to RHR quantiles in low and high serum ALB

eMaterials 1. Baseline examinations
