# Supplementary material for: Impact of Resting Heart Rate on Cardiovascular Mortality According to Serum Albumin Levels in a 24-year Follow-up Study on a General Japanese Population: NIPPON DATA80
Source: J Epidemiol. 2023 May 5;33(5):227–35. doi: 10.2188/jea.JE20210114 (PMC10043153; doi:10.2188/jea.JE20210114)
Supplement: Supplementary file 1 [file je-33-227-s001.pdf]

eTable 1. Baseline characteristics for participants

	Low albumin				High albumin			
Total (RHR quantiles)	Q1	Q2	Q3	Q4	Q1	Q2	Q3	Q4
Number of participants	1007	1218	896	1153	910	1228	797	1154
Age, years	56 (13)	54 (13)	53 (14)	54 (15)	47 (11)	45 (11)	46 (12)	46 (12)
Sex, male, %	52	38	30	30	67	52	42	37
Smoking, %	33	29	26	26	41	37	34	32
Drinking, %	39	38	35	32	59	51	45	42
Body mass index, kg/m^2	22 (2.9)	23 (3.1)	23 (3.5)	23 (3.5)	23 (2.9)	23 (2.9)	23 (3.1)	23 (3.4)
Total cholesterol, mmol/L	4.7 (0.8)	4.7 (0.9)	4.7 (0.9)	4.8 (0.8)	4.9 (0.8)	5.0 (0.8)	5.1 (0.9)	5.1 (0.9)
Non-fasting blood glucose, mmol/L	5.4 (1.3)	5.7 (1.9)	5.7 (1.8)	6.2 (2.3)	5.2 (1.4)	5.3 (1.3)	5.4 (1.3)	5.8 (1.8)
Systolic blood pressure, mm Hg	138 (23)	135 (22)	136 (23)	139 (22)	132 (20)	133 (20)	135 (20)	139 (21)
Hypertension treatment, %	15.1	14	11	9.6	9	9.5	8	10.3
Men	Q1	Q2	Q3	Q4	Q1	Q2	Q3	Q4
Number of participants	424	328	270	325	620	592	524	583
Age, years	59 (12)	58 (12)	57 (12)	58 (13)	45 (11)	45 (11)	45 (12)	46 (11)
Smoking, %	57.8	61.3	67.8	63.4	55.5	65.2	64.9	69.1
Drinking, %	68.2	70.4	68.5	71.7	76.8	77.5	77.5	78.0
Body mass index, kg/m^2	22 (2.8)	22 (2.6)	22 (2.8)	22 (3.2)	23 (2.7)	23 (2.6)	23 (2.8)	23 (3.0)
Total cholesterol, mmol/L	4.6 (0.7)	4.5 (0.8)	4.6 (0.8)	4.6 (0.9)	4.9 (0.8)	4.9 (0.8)	5.0 (0.8)	5.1 (0.9)
Non-fasting blood glucose, mmol/L	7.1 (1.7)	7.5 (2.6)	7.7 (1.8)	8.5 (3.2)	6.8 (1.8)	6.9 (1.3)	7.0 (1.8)	7.5 (2.1)
Systolic blood pressure, mm Hg	140 (23)	142 (21)	143 (24)	146 (24)	133 (19)	134 (19)	137 (19)	141 (18)
Hypertension treatment, %	14.9	17.1	11.1	13.8	6.3	7.4	5.7	9.3

Women	Q1	Q2	Q3	Q4	Q1	Q2	Q3	Q4
Number of participants	670	581	504	508	603	570	598	663
Age, years	54 (13)	53 (12)	52 (13)	52 (13)	49 (12)	48 (14)	48 (12)	47 (13)
Smoking, %	7.5	6.4	8.9	10.4	7.1	9.8	11.4	8.3
Drinking, %	21.6	18.8	19.2	15.2	20.2	23.0	21.6	19.6
Body mass index, kg/m^2	22.8 (3.0)	22.9 (3.5)	22.8 (3.6)	22.8 (3.7)	23.1 (3.2)	22.8 (3.2)	22.8 (3.4)	23.0 (3.6)
Total cholesterol, mmol/L	4.9 (0.9)	4.8 (0.8)	4.8 (0.9)	4.8 (0.9)	5.0 (0.9)	5.0 (0.8)	5.1 (0.8)	5.1 (0.9)
Non-fasting blood glucose, mmol/L	7.1 (1.7)	7.1 (1.8)	7.3 (2.3)	7.7 (2.4)	6.7 (1.4)	6.9 (1.3)	7.1 (1.5)	7.5 (2.2)
Systolic blood pressure, mm Hg	133 (22)	131 (21)	133 (22)	136 (21)	131 (22)	131 (21)	135 (20)	138 (22)
Hypertension treatment, %	14.8	11.5	10.9	8.7	14.6	8.9	9.7	11.0

Values reported as mean (standard deviation) unless otherwise noted.

eTable 2. Crude mortality rates and hazard ratios for CHD, stroke, cerebral infarction and cerebral hemorrhage mortality according to RHR quantiles in low and high serum ALB

CHD mortality		Low albumin				High albumin			
Total	RHR quantiles	Q1 (<62)	Q2 (62–68)	Q3 (69–77)	Q4 (>77)	Q1 (<62)	Q2 (62–68)	Q3 (69–77)	Q4 (>77)
	Number of participants	1007	896	910	797	1,218	1,153	1,228	1,154
	Person-years	21,936	31,589	20,661	30,361	20,863	31,879	17,253	29,582
	Number of event	33	27	37	29	34	21	18	20
	Crude mortality	1.5	0.9	1.8	1.0	1.6	0.7	1.0	0.7
	Hazard ratio								
	Model 1	1	1.03 [0.62,1.71]	1.51 [0.94,2.42]	1.46 [0.88,2.41]	1.38 [0.85,2.24]	1.08 [0.62,1.89]	0.92 [0.53,1.61]	0.89 [0.51,1.56]
	Model 2	1	0.95 [0.57, 1.59]	1.41 [0.88,2.27]	1.24 [0.75,2.07]	1.34 [0.82,2.20]	0.92 [0.52,1.61]	0.76 [0.43,1.34]	0.63 [0.35,1.12]
Men	RHR quantiles	Q1 (<60)	Q2 (60–66)	Q3 (67–73)	Q4 (>73)	Q1 (<60)	Q2 (60–66)	Q3 (67–73)	Q4 (>73)
	Number of participants	424	328	270	325	620	592	524	583
	Person-years	8,350	6,483	5,486	6,048	16,025	15,430	13,570	14,530
	No of event	13	10	13	15	21	13	9	14
	Crude mortality	1.6	1.5	2.4	2.5	1.3	0.8	0.7	1.0
	Hazard ratio								
	Model 1	1	1.03 [0.45,2.35]	1.59 [0.74,3.43]	1.74 [0.82,3.66]	1.91 [0.93,3.87]	1.06 [0.45,2.46]	1.07 [0.45,2.56]	1.37 [0.63,2.95]
	Model 2	1	0.92 [0.40,2.11]	1.50 [0.69,3.25]	1.35 [0.63,2.88]	1.89 [0.92,3.90]	0.86 [0.37,2.03]	0.89 [0.36,2.17]	0.95 [0.43,2.10]

Women	RHR quantiles	Q1 (<64)	Q2 (64–70)	Q3 (71–77)	Q4 (>77)	Q1 (<64)	Q2 (64–70)	Q3 (71–77)	Q4 (>77)
	Number of participants	670	581	504	508	603	570	598	663
	Person-years	16,065	14,380	12,081	11,818	15,936	15,022	15,555	17,340
	Number of events	23	17	18	17	14	9	7	12
	Crude mortality	1.4	1.2	1.5	1.4	0.9	0.6	0.5	0.7
	Hazard ratio								
	Model 1	1	0.96 [0.51,1.79]	1.11 [0.59,2.06]	1.13 [0.60,2.13]	0.91 [0.47,1.78]	0.70 [0.32,1.52]	0.47 [0.20,1.11]	0.78 [0.38,1.56]
	Model 2	1	0.96 [0.51,1.81]	1.11 [0.59,2.09]	1.04 [0.54,1.98]	0.94 [0.47,1.83]	0.66 [0.30,1.43]	0.40 [0.17,0.94]*	0.57 [0.28,1.17]
Stroke mortality		Low albumin				High albumin			
Total	RHR quantiles	Q1 (<62)	Q2 (62–68)	Q3 (69–77)	Q4 (>77)	Q1 (<62)	Q2 (62–68)	Q3 (69–77)	Q4 (>77)
	Number of participants	1,007	896	910	797	1,218	1,153	1,228	1,154
	Person-years	21,936	31,589	20,661	30,361	20,863	31,879	17,253	29,582
	Number of events	82	67	56	75	48	40	54	33
	Crude mortality	3.7	2.1	2.7	2.5	2.3	1.3	3.1	1.1
	Hazard ratio								
	Model 1	1	1.05 [0.76,1.45]	0.95 [0.67,1.34]	1.53 [1.12,2.10]	0.87 [0.60,1.24]	0.96 [0.66,1.42]	1.09 [0.77,1.55]	0.67 [0.44,1.00]
	Model 2	1	1.02 [0.74,1.41]	0.92 [0.65,1.30]	1.45 [1.05,2.00]*	0.91 [0.63,1.31]	0.90 [0.61,1.33]	1.01 [0.70,1.43]	0.55 [0.36,0.83]

Male	RHR quantiles	Q1 (<60)	Q2 (60–66)	Q3 (67–73)	Q4 (>73)	Q1 (<60)	Q2 (60–66)	Q3 (67–73)	Q4 (>73)
	Number of participants	424	328	270	325	620	592	524	583
	Person-years	8,350	6,483	5,486	6,048	16,025	15,430	13,570	14,530
	Number of events	46	39	23	37	21	27	19	19
	Crude mortality	5.5	6.0	4.2	6.1	1.3	1.7	1.4	1.3
	Hazard ratio								
	Model 1	1	1.15 [0.75,1.76]	0.81 [0.49,1.33]	1.22 [0.79,1.89]	0.66 [0.38,1.11]	1.02 [0.63,1.67]	0.81 [0.47,1.40]	0.63 [0.36,1.08]
	Model 2	1	1.09 [0.71,1.67]	0.75 [0.45,1.24]	1.13 [0.72,1.76]	0.70 [0.41,1.19]	0.96 [0.58,1.57]	0.78 [0.44,1.36]	0.54 [0.31,0.93]
Female	RHR quantiles	Q1 (<64)	Q2 (64–70)	Q3 (71–77)	Q4 (>77)	Q1 (<64)	Q2 (64–70)	Q3 (71–77)	Q4 (>77)
	Number of participants	670	581	504	508	603	570	598	663
	Person-years	16,065	14,380	12,081	11,818	15,936	15,022	15,555	17,340
	Number of events	33	33	25	44	21	25	26	17
	Crude mortality	2.1	2.3	2.1	3.7	1.3	1.7	1.7	1.0
	Hazard ratio								
	Model 1	1	1.31 [0.81,2.12]	1.04 [0.62,1.75]	1.94 [1.23,3.06]**	1.00 [0.58,1.73]	1.43 [0.85,2.41]	1.29 [0.77,2.17]	0.79 [0.44,1.43]
	Model 2	1	1.32 [0.81,2.15]	1.05 [0.62,1.77]	1.99 [1.25,3.15]**	0.98 [0.56,1.70]	1.38 [0.81,2.33]	1.22 [0.73,2.05]	0.63 [0.35,1.15]

Cerebral infarction mortality		Low albumin				High albumin			
Total	RHR quantiles	Q1 (<62)	Q2 (62–68)	Q3 (69–77)	Q4 (>77)	Q1 (<62)	Q2 (62–68)	Q3 (69–77)	Q4 (>77)
	Number of participants	1,007	896	910	797	1,218	1,153	1,228	1,154
	Person-years	21,936	31,589	20,661	30,361	20,863	31,879	17,253	29,582
	Number of events	59	39	34	47	31	21	24	17
	Crude mortality	2.7	1.2	1.6	1.5	1.5	0.7	1.4	0.6
	Hazard ratio								
	Model 1	1	0.84 [0.56,1.27]	0.80 [0.52,1.23]	1.33 [0.91,1.97]	0.85 [0.55,1.33]	0.81 [0.49,1.35]	0.74 [0.46,1.20]	0.51 [0.29,0.87]
	Model 2	1	0.82 [0.54,1.23]	0.78 [0.51,1.19]	1.27 [0.86,1.88]	0.87 [0.56,1.36]	0.74 [0.44,1.23]	0.65 [0.40,1.06]	0.41 [0.24,0.71]
Male	RHR quantiles	Q1 (<60)	Q2 (60–66)	Q3 (67–73)	Q4 (>73)	Q1 (<60)	Q2 (60–66)	Q3 (67–73)	Q4 (>73)
	Number of participants	424	328	270	325	620	592	524	583
	Person-years	8,350	6,483	5,486	6,048	16,025	15,430	13,570	14,530
	Number of events	35	27	14	22	15	16	6	8
	Crude mortality	4.2	4.2	2.6	3.6	0.9	1.0	0.4	0.6
	Hazard ratio								
	Model 1	1	1.06 [0.64,1.75]	0.62 [0.33,1.16]	0.96 [0.56,1.65]	0.68 [0.36,1.26]	0.91 [0.49,1.68]	0.39 [0.16,0.94]	0.38 [0.18,0.83]
	Model 2	1	0.97 [0.58,1.62]	0.57 [0.31,1.08]	0.85 [0.49,1.47]	0.68 [0.37,1.28]	0.77 [0.42,1.43]	0.35 [0.14,0.85]	0.28 [0.13,0.62]

Female	RHR quantiles	Q1 (<64)	Q2 (64–70)	Q3 (71–77)	Q4 (>77)	Q1 (<64)	Q2 (64–70)	Q3 (71–77)	Q4 (>77)
	Number of participants	670	581	504	508	603	570	598	663
	Person-years	16,065	14,380	12,081	11,818	15,936	15,022	15,555	17,340
	Number of events	22	17	13	29	13	13	13	9
	Crude mortality	1.4	1.2	1.1	2.5	0.8	0.9	0.8	0.5
	Hazard ratio								
	Model 1	1	0.99 [0.53,1.87]	0.78 [0.39,1.55]	1.83 [1.05,3.21]	0.99 [0.49,1.97]	1.18 [0.59,2.35]	0.97 [0.49,1.93]	0.66 [0.30,1.42]
	Model 2	1	0.96 [0.51,1.81]	0.81 [0.41,1.63]	1.94 [1.10,3.42]	0.94 [0.46,1.89]	1.11 [0.55,2.23]	0.94 [0.46,1.87]	0.53 [0.24,1.18]
Cerebral hemorrhage mortality		Low albumin				High albumin			
Total	RHR quantiles	Q1 (<62)	Q2 (62–68)	Q3 (69–77)	Q4 (>77)	Q1 (<62)	Q2 (62–68)	Q3 (69–77)	Q4 (>77)
	Number of participants	1,007	896	910	797	1218	1,153	1,228	1,154
	Person-years	21,936	31,589	20,661	30,361	20,863	31,879	17,253	29,582
	Number of events	13	18	14	15	10	9	14	11
	Crude mortality	0.6	0.6	0.7	0.5	0.5	0.3	0.8	0.4
	Hazard ratio								
	Model 1	1	1.81 [0.88,3.72]	1.52 [0.71,3.24]	1.98 [0.93,3.30]	0.94 [0.41,2.71]	1.10 [0.46,2.61]	1.53 [0.71,3.33]	1.25 [0.55,2.82]
	Model 2	1	1.81 [0.88,3.70]	1.43 [0.67,3.06]	1.76 [0.83,3.76]	1.01 [0.47,2.56]	1.14 [0.47,2.73]	1.53 [0.70,3.34]	1.09 [0.47,2.52]

Male	RHR quantiles	Q1 (<60)	Q2 (60–66)	Q3 (67–73)	Q4 (>73)	Q1 (<60)	Q2 (60–66)	Q3 (67–73)	Q4 (>73)
	Number of participants	424	328	270	325	620	592	524	583
	Person-years	8,350	6,483	5,486	6,048	16,025	15,430	13,570	14,530
	Number of events	6	9	6	10	4	6	5	9
	Crude mortality	0.7	1.4	1.1	1.7	0.2	0.4	0.4	0.6
	Hazard ratio								
	Model 1	1	2.01 [0.71,5.64]	1.66 [0.53,5.14]	2.49 [0.90,6.86]	0.84 [0.23,3.02]	1.45 [0.45,4.65]	1.36 [0.40,4.56]	2.00 [0.69,5.74]
	Model 2	1	1.95 [0.69,5.50]	1.59 [0.51,4.96]	2.40 [0.86,6.72]	1.03 [0.28,3.81]	1.70 [0.52,5.52]	1.56 [0.45,5.37]	2.31 [0.77,6.85]
Female	RHR quantiles	Q1 (<64)	Q2 (64–70)	Q3 (71–77)	Q4 (>77)	Q1 (<64)	Q2 (64–70)	Q3 (71–77)	Q4 (>77)
	Number of participants	670	581	504	508	603	570	598	663
	Person-years	16,065	14,380	12,081	11,818	15,936	15,022	15,555	17,340
	Number of events	6	10	6	7	4	5	7	4
	Crude mortality	0.4	0.7	0.5	0.6	0.3	0.3	0.5	0.2
	Hazard ratio								
	Model 1	1	2.15 [0.78,5.92]	1.43 [0.46,4.43]	1.79 [0.60,5.34]	0.91 [0.25,3.24]	1.34 [0.40,4.41]	1.75 [0.58,5.22]	0.92 [0.25,3.27]
	Model 2	1	2.20 [0.79,6.10]	1.23 [0.39,3.91]	1.57 [0.52,4.77]	0.94 [0.26,3.36]	1.25 [0.37,4.14]	1.45 [0.47,4.41]	0.59 [0.16,2.17]

Model 1 is adjusted for age and gender (in gender specific models gender is not adjusted)

Model 2 is adjusted for gender (in gender specific models gender is not adjusted), age, body mass index, blood glucose, systolic blood pressure, hypertension treatment, total-cholesterol, smoking status, and alcohol drinking status

eMaterials 1. Baseline examinations

Details of the baseline examination are described in previous studies.[1-3] The baseline characteristic surveys were conducted by local public health centers. The participants' blood pressure (BP) was obtained by trained technicians using a standard mercury sphygmomanometer after at least 5 min of rest. The public health nurses collected information on smoking and drinking and medical history. All blood samples were obtained without fasting. Then, they were centrifuged within 60 min after collection. The samples were stored at −70°C until analyses. The ALB and total cholesterol (TCH) levels were analyzed in an autoanalyzer (SMA12; Technicon, Tarrytown, the USA) with the Lieberman-Burchard direct method for TCH levels and the bromocresol-green method for ALB levels at a specific laboratory (formerly, Center for Adult Diseases, Osaka; current name, Osaka Center for Cancer and Cardiovascular Disease Prevention). The laboratory is a member of the Cholesterol Reference Method Laboratory Network (CRMLN),[4] and measurements were certified in the Lipid Standardization Program administered by the Center for Disease Control and Prevention, Atlanta. Two researchers individually interpreted ECG recordings according to the Minnesota code, and RHR was determined by measuring three consecutive intervals between R waves on the 12-lead ECG after the participant had enough rest.[2, 3] The Minnesota codes were as follows: code 8–3–3, atrial fibrillation; code 8–1–1, frequent supraventricular premature beats; and code 8–1–2, ventricular premature beats. The blood glucose level was measured in milligrams per deciliter (mg/dL), and it was converted into millimoles per liter (mmol/L) ([0.047 × (glucose concentration in mg/dL)] − 0.541), as published in a previous study.[5]

Follow-up surveys

In this study, to examine the relationship between RHR and cause-specific mortality, the follow-up period was extended to 29 years.[3] All participants were followed-up based on mortality record until 2009 under the Family Registration Law in the municipalities where participants registered. With permission from the Management and Coordination Agency of the Japanese government, data from the National Vital Statistics were used to identify causes of death. All death records were coded according to the 9th International Classification of Disease (ICD-9) until the end of 1994 and ICD-10 from the start of 1995. The corresponding ICD-9 and ICD-10 codes used in the current study were as follows: CVD mortality, 393–459 (ICD-9) and I00–I99 (ICD-10); CHD mortality, 410–414 (ICD-9) and I20–I25 (ICD-10); and stroke mortality, 430–438 (ICD-9) and I60–I69 (ICD-10). Death with causes other than CVD was defined as non-CVD mortality.

References

1. Hisamatsu T, Miura K, Ohkubo T, Yamamoto T, Fujiyoshi A, Miyagawa N, Kadota A, Takashima N, Okuda N, Yoshita K, Kita Y, Murakami Y, Nakamura Y, Okamura T, Horie M, Okayama A, Ueshima H; NIPPON DATA80 Research Group. High long-chain n-3 fatty acid intake attenuates the effect of high resting heart rate on cardiovascular mortality risk: A 24-year follow-up of Japanese general population. Journal of Cardiology, 2014. 64(3): p. 218-224.

2. Okamura T, Hayakawa T, Kadowaki T, Kita Y, Okayama A, Elliott P, Ueshima H; NIPPON DATA80 research group. *A combination of serum low albumin and above-average cholesterol level was associated with excess mortality.* J Clin Epidemiol, 2004. **57**(11): p. 1188-95.
3. Okamura T, Hayakawa T, Kadowaki T, Kita Y, Okayama A, Elliott P, Ueshima H; NIPPONDATA80 Research Group. *Resting heart rate and cause-specific death in a 16.5-year cohort study of the Japanese general population.* Am Heart J, 2004. **147**(6): p. 1024-32.
4. Nakamura, M., S. Sato, and T. Shimamoto, *Improvement in Japanese clinical laboratory measurements of total cholesterol and HDL-cholesterol by the US Cholesterol Reference Method Laboratory Network.* J Atheroscler Thromb, 2003. **10**(3): p. 145-53.
5. Kadowaki S, Okamura T, Hozawa A, Kadowaki T, Kadota A, Murakami Y, Nakamura K, Saitoh S, Nakamura Y, Hayakawa T, Kita Y, Okayama A, Ueshima H; NIPPON DATA Research Group. *Relationship of elevated casual blood glucose level with coronary heart disease, cardiovascular disease and all-cause mortality in a representative sample of the Japanese population. NIPPON DATA80.* Diabetologia, 2008. **51**(4): p. 575-82.
